# Lyme borreliosis in Belgium: a cost-of-illness analysis

**DOI:** 10.1186/s12889-022-14380-6

**Published:** 2022-11-28

**Authors:** Laurence Geebelen, Brecht Devleesschauwer, Tinne Lernout, Katrien Tersago, Yves Parmentier, Herman Van Oyen, Niko Speybroeck, Philippe Beutels

**Affiliations:** 1 Scientific Directorate of Epidemiology and Public Health, Sciensano, Brussels, Belgium; 2 Institute of Health and Society (IRSS), Université Catholique de Louvain, Woluwe-Saint-Lambert, Belgium; 3 Department of Translational Physiology, Infectiology and Public Health, Ghent University, Merelbeke, Belgium; 4 Flemish Agency for Care and Health, Brussels, Belgium; 5 Technical Cell (CT-TC), National Institute for Health and Disability Insurance (INAMI-RIZIV), Brussels, Belgium; 6 Department of Public Health and Primary Care, Ghent University, Ghent, Belgium; 7 Centre for Health Economics Research & Modelling Infectious Diseases (CHERMID), Vaccine & Infectious Disease Institute, Faculty of Medicine & Health Sciences, University of Antwerp, Wilrijk, Belgium

**Keywords:** Economic cost, Lyme borreliosis, Erythema migrans, Disseminated Lyme borreliosis, Late Lyme borreliosis, Belgium

## Abstract

**Background:**

Lyme borreliosis (LB) is the most common tick-borne disease in Europe and North America, yet its economic burden remains largely unknown. This study aimed to estimate the economic cost associated with the different clinical manifestations of LB in Belgium.

**Methods:**

An incidence approach and societal perspective were used to estimate the total cost-of-illness for LB in Belgium. Costs were calculated for patients with erythema migrans (EM) or disseminated/late LB, including patients who developed post-treatment Lyme disease syndrome (PTLDS). Direct medical, direct non-medical (transportation & paid help) and indirect non-medical costs (productivity losses) were included in the analysis. Ambulatory cost data were collected through a prospective cohort study from June 2016 to March 2020, in which patients with LB were followed up 6 to 12 months after diagnosis. Hospitalization costs were retrieved from the Minimal Clinical Data registry, a mandatory registry for all Belgian hospitals, linked to the Minimal Financial Data registry. Costs were expressed in 2019 euros.

**Results:**

The total annual cost associated with clinical manifestations of LB in Belgium was estimated at €5.59 million (95% UI 3.82–7.98). Of these, €3.44 million (95% UI 2.05–5.48) or 62% was related to disseminated/late LB diagnoses and €2.15 million (95% UI 1.30–3.26) to EM. In general, direct medical costs and productivity losses accounted for 49.8% and 46.4% of the total costs, respectively, while direct non-medical costs accounted for only 3.8%. The estimated mean costs were €193 per EM patient and €5,148 per disseminated/late LB patient. While patients with PTLDS seemed to have somewhat higher costs compared to patients without PTLDS, the number of patients was too small to have representative estimates.

**Conclusions:**

We estimate the total annual direct medical costs, direct non-medical and indirect non-medical costs associated with LB to exceed €5.5 million per year, almost evenly distributed between EM (40%) and disseminated/late LB (60%). EM costs 26 times less per patient but occurs also 16 times more frequently than disseminated/late LB. The cost burden remains limited by comparison to other infectious diseases due to the relative lower incidence.

**Supplementary Information:**

The online version contains supplementary material available at 10.1186/s12889-022-14380-6.

## Introduction

Lyme borreliosis (LB) is the most common tick-borne disease in Europe. An erythema migrans (EM), a red expanding rash at the site of the tick bite, is one of the first and often the only symptom present. If left untreated the infection can disseminate causing more severe disease such as Lyme neuroborreliosis (LNB), Lyme arthritis (LA), Lyme carditis or acrodermatitis chronica atrophicans (ACA) [1, 2]. Even after appropriate antibiotic treatment, persisting non-specific symptoms such as fatigue, widespread musculoskeletal pain or cognitive difficulties have been reported by a subset of patients. When these symptoms persist for 6 months or more and are of such severity that they impact daily activities, this is often referred to as post-treatment Lyme disease syndrome (PTLDS) [3]. In Belgium, the annual incidence of LB has been estimated at 103 per 100,000 inhabitants (95% UI 87–120) for the period 2015–2017 [4]. A study on the prioritization of communicable diseases for future surveillance, performed in 2018, categorized it in its “high priority” diseases group [5]. Yet, the exact burden remains unknown. Cost-of-illness studies are an important part of the assessment of the impact of a disease on the healthcare system and society. They provide essential information for cost-effectiveness and budget-impact analyses, which in turn can be used for policy making on budget allocation and on prioritizing interventions that prevent or control disease burden. Studies assessing the economic burden of LB are limited, both in North-America and Europe [6, 7]. To the best of our knowledge, no such studies have been published yet for Belgium. The aim of this cost-of-illness study was to assess the economic burden of LB in Belgium, taking into account different clinical manifestations and PTLDS.

## Methods

The total cost of LB was estimated through an incidence-based costing approach for patients with (i) an EM (including multiple EM) or (ii) disseminated/late LB, including patients who later developed PTLDS. A societal perspective, which considers costs for the national healthcare insurer, the patients and their employers, was taken. Direct medical costs (ambulatory [aka outpatient] and hospital [aka inpatient] care), direct non-medical costs (transportation) and indirect non-medical costs (productivity losses due to sick leave) were included using different data sources. Mean costs were extrapolated to the population level using LB incidence estimates [4] and the number of hospitalizations over multiple years to account for yearly fluctuations.

### Direct medical costs

#### Ambulatory care

The current study is part of a larger project in which a prospective cohort study, “HUMTICK”, was set up to collect data on LB in Belgium. The methodology of this study has been described elsewhere [8]. Briefly, between June 2016 and December 2019, adult patients with an EM or disseminated/late LB (LNB, LA, Lyme carditis or ACA) were included in the study through a network of general practitioners and eight Belgian hospitals, respectively. The cohorts were followed up and patients who developed PTLDS were identified based on the case definition proposed by the Infectious Disease Society of America (IDSA) [3]. The results of the PTLDS analysis are published elsewhere [9]. A total of 108 EM patients and 15 disseminated/late LB patients, prospectively recruited for the PTLDS baseline analysis, were included in the current cost analysis. Six EM and three disseminated/late LB patients fulfilled the complete PTLDS definition. Three EM patients and two disseminated/late LB patients had a missing value for PTLDS, and the information for these cases was imputed [9]. Since for each of these patients, either all imputations or the majority of the imputations led to the conclusion of “no PTLDS”, these five patients were assigned to the “no PTLDS” groups for the purpose of our analysis.

Data on volumes of resource use, more specifically consultations, medication, but also travel expenses, non-medical paid help, informal care and productivity losses (see further) were collected through patient questionnaires. All patients were asked to fill in a questionnaire at diagnosis, and then at 1 and 3 months post-diagnosis and at 6 months post-treatment. If included in the first years of the study, longer follow-up was possible with a questionnaire at 12 and possibly 24 months post-treatment. A cost diary was provided for patients’ personal use. In the current analysis, costs occurring until six months after treatment were included for EM patients and disseminated/late LB patients and costs occurring until 12 months for patients with PTLDS. In the end, the number of patients with data at 24 months was too limited to include this time point for the cost calculations in the sections below.

The number of LB serological tests performed for EM patients at diagnosis or during the first 2 months after diagnosis, although not required, was estimated from data of the network of sentinel general practices (SGP) which follows up the incidence of EM in Belgium [4]. When ELISA and/or immunoblot were performed, it was assumed to include both IgM and IgG testing. For disseminated/late LB, the specialists enlisting patients in the cohort study provided this information. For hospitalized patients in the cohort study, costs occurring during these hospitalizations, e.g., part of treatment, laboratory testing, were removed as these are calculated based on separate data (see further). Intravenous treatment was considered to be provided during (day) hospitalization only.

Unit prices in euro (€) for the reference year 2019 were used. For serological tests and consultations with reimbursed care providers (general practitioner (GP), specialist, physiotherapist, psychologist), unit prices were obtained from the National Institute of Health and Disability Insurance [10, 11] (Additional file 1, Table S1 and S2). For alternative therapists (osteopath, homeopath, chiropractor) prices are not fixed in Belgium. Based on reports of the Belgian Health Care Knowledge Centre (KCE) [12, 13] and the website of the Belgian professional association of osteopathy [14], an amount of €50 per consult was attributed (Additional file 1, Table S1). Prices of medication were retrieved from the Belgian Centre for Pharmacotherapeutic Information [15]. For antibiotics, and in line with the Belgian guidelines for economic evaluations [16], the cheapest brand was counted as pharmacists in Belgium are obliged to substitute (Additional file 1, Table S3). For food supplements, not described by the BCPI, prices were obtained from the website of the brand itself or – if not available – online pharmacies. If no brand was given for a product, a random value based on a uniform distribution between the minimum and maximum price of brands available online was drawn. In general, the smallest package size containing the number of tablets used by the patient was counted. If the number of tablets was not specified, the smallest box on the market was counted.

#### Hospitalizations

Data on the costs of hospitalizations for LB were retrieved from the Minimal Clinical Data registry (MCD) linked to the Minimal Financial Data registry (MFD) for the year 2016. The MCD is a registry mandatory for all Belgian hospitals containing information such as the primary and secondary diagnosis, the length of stay and patient demographics, on both classical hospitalizations (at least one overnight) and day hospitalizations. Diagnosis coding in the MCD registry is, since 2015, based on the International Statistical Classification of Diseases—10^th^ revision—Clinical Modification (ICD-10-CM). Hospitalizations were identified based on the primary diagnosis being LB, corresponding to the ICD-10-CM LB codes listed in Table 1.

**Table 1:** ICD-10-CM codes Lyme Borreliosis, 2016, Belgium

A69.20	Lyme disease unspecified
A69.21	Meningitis due to Lyme disease
A69.22	Other neurologic disorders in Lyme disease
A69.23	Arthritis due to Lyme disease
A69.29	Other conditions associated with Lyme disease
L90.4	Acrodermatitis chronica atrophicans

https://www.health.belgium.be/en/node/27415

For the classical hospitalizations, hospital specific 100% day prices for the year 2019 were multiplied by the number of inpatient days. Other hospital costs available in the MFD registry for 2016 were converted to the reference year 2019 by applying an inflation of 5.16% (Health index 2016–2019) [17]. The cost of drugs administered during overnight hospitalizations consists of three components: a lump sum per admission, a lump sum by day (€0.62) and a cost per drug administered. For day hospitalizations (hospital stay without night), there is no lump sum for medication. Day costs, medication and radiopharmaceuticals, include both reimbursed and non-reimbursed costs. In contrast, costs for laboratory diagnostics, medical acts or implants include reimbursed costs only since information on non-reimbursed costs is not available in the MFD. Costs counted included both LB specific (e.g. antibiotics, LB serology) and non-LB specific costs. Supplements for a single room, costs for food, ambulance, etc. could not be counted as this information was not available in the MCD-MFD data. Such supplements tend to be highly variable between hospitals and depends also on the socioeconomic background of patients [18, 19].

### Direct non-medical costs

For travel expenses related to both ambulatory care and hospitalizations, volumes reported by the patients in the prospective cohort study were used. The number of kilometers travelled by private transport were multiplied with the Belgian kilometric allowance 2019 (€0.3653). If public transport was used, the ticket price was counted. Paid non-medical help (household cleaning, childcare, yard maintenance) was valued using expenditures reported by the patients. Informal care from friends or family was measured but not attributed a monetary value, following Belgian guidelines for economic evaluations [16].

### Indirect non-medical costs

The costs of productivity losses (work absenteeism) were estimated using the human capital approach (i.e., without friction period), i.e., the number of hours of sick leave were multiplied with the gross national average hourly labor cost being €40.5 in 2019 [20]. Hours of ambulatory productivity loss (not related to hospitalization) were estimated based on the prospective cohort study responses. In accordance with the Belgian Labour Act, one week of sick leave was counted as 38 h productivity losses for fulltime work and 19 h for part-time work. Hours of productivity losses due to hospitalization was calculated based on the mean length of hospital stay for cases < 65 years old in the MCD-MFD data, an employment rate of 65.3% for the Belgian population aged 15–65 years (of which 72% fulltime, 13% 4/5^th^, 10% halftime and 4.9% other part-time work counted as halftime) [21, 22] and, following KCE guidelines, assuming 18.8 working days per month with 7.6 working hours per day [16]. For day hospitalization, half a day productivity loss was counted (estimation based on the duration of IV treatment administration). For children it was assumed that one parent takes sick leave.

### Number of LB cases in Belgium

To extrapolate to the population level, the estimated average ambulatory costs by group were multiplied with (i) the total number of EM cases and (ii) the total number of disseminated/late LB cases in Belgium, based on the average annual incidence reported by Geebelen et al. (2019) for the period 2015─2017 [4]. In these, the disseminated/late LB group included LNB, LA, Lyme carditis and ACA but also borrelial lymphocytoma and ocular manifestations (not included in the prospective cohort study). A total population size of 11,462,024 was applied (Belgium, mid-year 2019) [23]. The PTLDS patients and their costs were included in the cost estimation as part of the group from which they emanated (group (i) or (ii)). For the hospitalizations, the annual mean number of hospital admissions for the period 2016–2018 was used. As there are no specific codes for EM in the ICD-10-CM coding system, and as EM is not expected to cause hospitalization when occurring on its own, the hospitalization costs were assigned to the more severe manifestation group (ii) disseminated/late LB.

### Data analysis

All analyses were conducted in R version 3.6.3 [24]. For different cost categories within the ambulatory data and hospital data, uncertainty was estimated performing a non-parametric bootstrap procedure with 10,000 replications to calculate 10,000 bootstrapped means (distributions). The bootstrapped means of ambulatory costs for (i) EM patients and (ii) disseminated/late LB patients, more specifically the bootstrapped means of the total direct medical costs, the direct non-medical (travel expenses, paid non-medical help) and the indirect non-medical costs (productivity losses), were multiplied with the number of cases in Belgium on which uncertainty was propagated using 10,000 Monte Carlo simulations following a uniform distribution defined by the mean minimum and mean maximum incidence estimate for the period 2015─2017 [4]. Bootstrapped means of direct medical and indirect non-medical costs related to (day-)hospitalizations were multiplied with the mean number of stays for the period 2016─2018. The results for the different cost categories (distributions) were further summed by patient group to calculate total costs for Belgium capturing the uncertainty on the input parameters in the final outputs. All results were summarized by the mean and 95% uncertainty interval (UI) defined as the 2.5^th^ and 97.5^th^ percentile of the bootstrap or uncertainty distribution. Additional file 2 provides an overview of the estimated median costs by patient, calculated by performing a non-parametric bootstrap procedure with 10,000 replications to calculate 10,000 bootstrapped medians, summarized by the mean and uncertainty interval of the bootstrap distribution, as described above. Unless specified differently earlier (e.g. medication), missing values were imputed with the median of the observed values in the same group at the same time point or multiple time points combined if n observations < 5. When patients reported a range instead of an exact value, the mean of the range was used.

## Results

### Direct medical costs

#### Ambulatory care

The mean ambulatory direct medical cost was estimated at €124 (95% UI 90.3–165)) for EM patients (i) and €516 (95% UI 350–731) for disseminated/late LB patients (ii), including patients who developed PTLDS in both groups (Table 2). Estimates for patients without or with PTLDS separately are shown in Table 2. Ambulatory direct medical costs were highest for patients with disseminated/late LB at a mean of €524 (95% UI 327–787) and €478 (95% UI 262–723) for those without and with PTLDS, respectively. Within the EM group, the mean ambulatory direct medical cost was almost twice as high for patients with PTLDS (€214, 95% UI 87.7–382) compared to without PTLDS (€118, 95% UI 85.7–162). The latter was mainly due to a difference in Over-The-Counter (OTC) medication (mean difference €65.2) followed by consultations (mean difference €30.1).

**Table 2:** Estimated mean ambulatory cost per patient, expressed in 2019 euros, for the different manifestation groups of Lyme borreliosis in the prospective cohort study (HUMTICK), 2016─2020, Belgium. Mean and 95% uncertainty intervals of the bootstrap distribution of the mean

	**Unit cost**	**(i) Erythema migrans**			**(ii) Disseminated/late LB**		
		**No PTLDS**	**PTLDS**	**All**	**No PTLDS**	**PTLDS**	**All**
**Nb pts in cohort**		**102**	**6**	**108**	**12**	**3**	**15**
**Direct medical costs**							
**Total**		**€118 (85.7–162)**^**a**^	**€214 (87.7–382)**^**a**^	**€124 (90.3–165)**^**a**^	**€524 (327–787)**	**€478 (262–723)**	**€516 (350–731)**
**Consultations**		**€61.5 (41.1–89.1)**	**€91.6 (39.4–146)**	**€63.1 (43.1–89.1)**	**€352 (255–452)**	**€283 (85.3–382)**	**€338 (252–426)**
GP	€26.3	€40.6 (34.8–47.6)	€47.9 (30.6–65.7)	€41 (35.3–47.6)	€105 (72.2–142)	€105 (0–158)	€105 (73.6–139)
Specialist^b^	€26.3–60.0	€11.4 (2.5–23.9)	€15.4 (0–46.5)	€11.6 (3–23.4)	€144 (107–186)	€115 (85.3–140)	€138 (109–173)
Emergency^c^	€34.9	€0 (0–0)	€0 (0–0)	€0 (0–0)	€26.2 (14.5–37.8)	€46.4 (0–105)	€30.4 (16.3–44.2)
Alternative^d^	€50	€5.4 (0.5–13.2)	€16.9 (0–50)	€6.1 (0.9–13.9)	€45.9 (12.5–87.5)	€16.7 (0–50)	€40.1 (13.3–73.3)
Others^e^	€22.3–60.0	€4.1 (0–11.7)	€11.3 (0–33.4)	€4.5 (0–12.3)	€30.5 (0–63.8)	€0 (0–0)	€24.3 (0–52.5)
**Medication**		**€39 (26.4–55.1)**	**€106 (26.6–227)**	**€42.8 (28.9–59.4)**	**€159 (51.9–346)**	**€175 (65.8–341)**	**€163 (66.2–315)**
Antibiotics at T0		€19.5 (17.9–21.1)	€15.1 (12–18.3)	€19.2 (17.7–20.8)	€11.4 (2.9–21.7)	€24.9 (0–41.2)	€14 (5–23.3)
Prescription		€4.2 (0.3–11.2)	€10.7 (0–27.3)	€4.5 (0.6–11.6)	€26.1 (12.9–39.9)	€46.5 (13.4–75.8)	€30.2 (17.4–43.6)
Over the counter		€15.4 (4.6–29.3)	€80.6 (8.9–202)	€19.1 (6.9–34.3)	€122 (18.6–306)	€104 (0–294)	€119 (22.7–273)
**LB serology**		**€16.3 (13.9–18.7)**^**f**^	**€16.3 (13.9–18.7)**^**f**^	**€16.3 (13.9–18.7)**^**f**^	**€9.8 (0–24.2)**^**g**^	**€19.8 (0–59.4)**^**g**^	**€11.6 (0–23.6)**^**g**^
**Other**^**h**^		**€1.5 (0–3.9)**	**€0 (0–0)**	**€1.4 (0–3.7)**	**€3.3 (0–10)**	**€0 (0–0)**	**€2.7 (0–8)**
**Indirect non-medical costs**							
**Ambulatory**^**i**^	€40.5/hr	**€62.7 (6–145)**	**€70.5 (0–213)**	**€63.9 (7.1–141)**	**€2,432 (286–5,724)**	**€3,341 (0–5,604)**	**€2,624 (708–5,345)**

*Nb* Number, *LB* Lyme borreliosis, *PTLDS* Post-treatment Lyme disease syndrome, *SGP* sentinel general practices, *GP* General practitioner, *EM* Erythema migrans

^a^The bootstrapped means of the total direct medical costs in the cohort study was summed with the bootstrapped means of LB serology in SGP data 2015─2017 and the resulting distribution summarized by the mean and 95% uncertainty interval

^b^Neurologist, rheumatologist, infectious disease specialist, dermatologist, gastroenterologist, orthopedist, radiologist (consult + forfait by prescription)

^c^Visit and care included only (no medication/performances); price visit without referral by a GP, on a weekday and care provided by a specialist in emergency medicine

^d^Homeopath, osteopath, chiropractor

^e^Physiotherapist, psychologist

^f^Based on SGP data 2015─2017: at diagnosis or in the first 2 months after, ELISA (1st tier test) was performed in 39.7% and Immunoblot (2nd tier test) in 19.0% of EM cases with available information (*n* = 348), no differentiation could be made between patients without or with PTLDS

^g^Only includes testing performed in patients that were not hospitalized and did not have Lyme neuroborreliosis (*n* = 3), as the latter needs cerebrospinal fluid testing, all tests in these patients and hospitalized patients were expected to be performed during (day-)hospitalization

^h^Scans: CT-scan, MRI or echo in ambulatory patient

^i^Productivity losses taking into account part-time work in the cohorts

#### Hospitalizations

In 2016, there were 286 classical hospital admissions (for at least one night) for 269 patients in total, with LB as a primary diagnosis. The mean duration of a hospital stay equaled 5.6 days (95% UI 4.8–6.5 days) corresponding to a mean direct medical cost of €4,159 per hospital admission (Table 3). Within the direct medical cost for overnight hospitalizations, hospital day price was the most important cost at a mean of €3,036 (73%), followed by the medical acts (20%), medication (4%) and laboratory tests (2%). LB serology was performed during 79% of stays and at least one antibiotic treatment, possibly for LB, was given during 67% of stays. Pain killers were given during 41% of stays.

**Table 3:** Estimated mean costs per classical hospitalization and day hospitalization, 2016 euros converted to 2019 euros, Belgium. Mean and 95% uncertainty intervals of the bootstrap distribution of the mean

	**Mean unit cost**	**Cost per hospital stay**
		**Mean (95% UI)**
**Overnight hospital stays (*n* = 286)**		
**Total direct medical costs**		**€4,159 (3,604–4,787)**
**100% day cost**	**€540**	** €3,036 (2,559–3,593)**
**Medication**		** €170 (140–213)**
Lump sum per admission	€95.7	€94.7 (92–97.4)
Lump sum per day^a^	€0.62	€3.5 (3–4)
Antibiotics^b^		€13.5 (11.1–16.2)
Pain killers		€1 (0.7–1.4)
Others		€56.8 (28.7–99.7)
**Laboratory tests**		** €101 (89.1–113)**
LB serology		€13.7 (12–15.3)
Others		€87 (76–98.4)
**Medical acts**		** €841 (771–916)**
Clinical biology		€228 (204–257)
Permanence, examinations		€204 (189–221)
Medical imaging		€199 (180–219)
Others^c^		€210 (180–242)
**Implants**^**d**^		** €5.5 (2.5–9.1)**
**Radiopharmaceuticals**		** €6.7 (2.9–11.1)**
**Indirect non-medical cost (productivity loss)**^**e**^	**€40.5/hr**	**€399 (348–452)**
**Day hospital (*n* = 613)**		
**Total direct medical costs**		**€50.5 (35.7–69.7)**
**Indirect non-medical cost (productivity loss)**^**e**^	**€40.5/hr**	**€29.8**

^a^Lump sum per day: forfait for reimbursed medicines, charged to the patient by day even if such medicines have not been used

^b^Antibiotics probably related to Lyme borreliosis: Ceftriaxon, Doxycycline, Amoxicilline, Clarithromycine, cefuroxime, azithromycine, cefotaxim, ampicilline cefepime, cefazoline and flucloxacilline

^c^Including internal medicine, revalidation, surgery, night/weekend supplements and others

^d^Mainly catheter

^e^Based on mean bootstrapped days of stay (< 65 yrs olds) * proportion hospitalizations < 65 yrs old * proportion of < 65 yrs olds working *proportion working days *7.6 h * €40.5. For day hospitalizations, the number of days was multiplied with 0.5 as half a day of sick leave was counted

MCD-MFD data were available for 613 out of 684 days for day hospitalizations with LB as a primary diagnosis in 2016. As these concerned 68 unique patients, they each had on average 9 day hospitalisations. The mean direct medical cost for one day hospitalization equaled €50.5. The majority of patients (*n* = 57/68) received intravenous antibiotic treatment and medication caused 53% of costs.

### Direct non-medical costs

The estimated mean travel expenses related to ambulatory care or hospitalization equaled €5.0 (95% UI 1.0–11.5) in EM patients and €190 (95% UI 92–297) in disseminated/late LB patients. Within the EM group, mean costs were higher in patients with PTLDS (€16.2, 95% UI 0.9–42.1) compared to no PTLDS (€4.4, 95% UI 0.6–10.7). For disseminated/late LB, costs were lower for patients with PTLDS (€97.2, 95% UI 0–219) than no PTLDS (€213, 95% UI 94.4–341), but the number of patients in the disseminated/late LB group and PTLDS groups were low.

Only 6/108 (5.6%) EM patients reported the use of informal care of family or friends compared to 8/15 (53.3%) of disseminated/late LB patients. The mean number of hours of help received was highest for EM patients with PTLDS (18.8, 95% UI 0.33–48.50), followed by disseminated/late LB without PTLDS (12.7, 95% UI 4.7–21.7), disseminated/late LB with PTLDS (2.01, 95% UI 0.0–6.0) and EM patients without PTLDS (1.21, 95% UI 0.0–3.4). Paid non-medical help was only reported by one patient with disseminated/late LB without PTLDS, declaring a cost of €600 (gardener), leading to a mean cost of €40.8 (95% UI 0.0–120) for all disseminated/late LB patients (*n* = 15).

### Indirect non-medical costs

The estimated mean costs for productivity losses, not related to hospitalization, by patient group are shown in Table 2. The cost was highest for disseminated/late LB patients with PTLDS, at a mean of €3,341 (95% UI 0–5,604) corresponding to 10.9 working days, followed by disseminated/late LB without PTLDS (€2432; 95% UI 286–5724; 7.9 days), EM with PTLDS (€71; 95% UI 0–213; 1.75 h) and EM without PTLDS (€62.7; 95% UI 6–145; 1.55 h). The mean cost for productivity losses related to hospitalizations (group ii) was estimated at €399 (95% UI 348–452) per overnight hospital admission and €29.8 per day hospitalization (Table 3).

### Total LB cost in Belgium

Table 4 shows the mean number of LB cases, the mean number of hospitalizations and the total cost estimates for the different clinical manifestations of LB for the whole of Belgium per year. The total annual cost, including ambulatory care, hospital care, travel expenses, non-medical help and productivity losses, equaled €5.59 million (95% UI 3.82–7.98). Of these, €3.44 million (95% UI 2.05–5.48) or 62% was related to disseminated/late LB diagnoses and €2.15 million (95% UI 1.30–3.26) or 38.4% to EM. In general, direct medical costs and productivity losses accounted for 49.8% and 46.4% of the total costs, respectively. Direct non-medical costs accounted for 3.8%. The proportion of costs for productivity losses was higher in disseminated/late LB patients (54.6%) than in EM patients (33.2%). Out of the direct medical costs in all patients, 38.0% was related to hospitalizations or day hospitalizations, whereas within the disseminated/late LB patient group this was 75.1%. The majority of direct medical costs were reimbursed, namely 75.5%, yet not all non-reimbursed costs for hospitalizations could be counted and the proportion reimbursed depends on the patient’s insurance. A division between costs reimbursed by the health insurance system and non-reimbursed costs for the patients themselves is provided in Additional file 3 (Table S7).

**Table 4:** Incidence number of cases and total costs for Lyme borreliosis in Belgium. Total costs and 95% uncertainty interval

	**(i) Erythema migrans**	**(ii) Disseminated/late LB**	**Total**
**Nb cases**	**11,168 (9,417–12,954)**	**673 (519–867)**	**11,840 (9,976–13,732)**
**Nb overnight hospitalizations**	**0**	**248**^**a**^	**248**
**Nb day hospitalizations**	**0**	**539**^**b**^	**539**
**Direct medical costs**	**€1,379,838 (954,446–1,940,745)**	**€1,406,501 (1,210,178–1,645,284)**	**€2,786,338 (2,278,144–3,411,333)**
Ambulatory	€1,379,838 (954,446–1,940,745)	€346,965 (215,580–529,594)	€1,726,803 (1,241,868–2,336,606)
Hospital	€0	€1,032,324 (894,388–1,188,219)	€1,032,324 (894,388–1,188,219)
Day hospital	€0	€27,211 (19,219–37,548)	€27,211 (19,219–37,548)
**Direct non-medical costs**	**€56,338 (11,223–130,773)**	**€155,000 (70,394–263,255)**	**€211,338 (108,891–341,204)**
Travel expenses	€56,338 (11,223–130,773)	€127,563 (58,472–215,098)	€183,901 (95,605–298,909)
Paid non-medical help	€0 (0–0)	€27,437 (0–90,998)	€27,437 (0–90,998)
**Indirect non-medical costs (productivity loss)**	**€713,532 (84,747–1,595,890)**	**€1,880,849 (576,266–3,858,549)**	**€2,594,380 (1,046,331–4,805,398)**
Ambulatory	€713,532 (84,747–1,595,890)	€1,765,836 (464,016–3,739,760)	€2,479,367 (930,220–4,687,748)
Hospital	€0	€98,973 (86,376–112,134)	€98,973 (86,376–112,134)
Day hospital	€0	€16,040 (16,040–16,040)	€16,040 (16,040–16,040)
**Total costs**	**€2,149,707 (1,299,782–3,262,206)**	**€3,442,349 (2,048,208–5,480,688)**	**€5,592,056 (3,816,810–7,979,428)**
**Total cost per patient**	**€193 (121–284)**	**€5,148 (3,091–7,911)**	**€473 (333–654)**

*Nb* Number, *LB* Lyme borreliosis

^a^Mean of 268, 217 and 234 classical hospitalizations (overnight) in 2016, 2017 and 2018, respectively, corrected for the Belgian population mid-year 2019 (*n* = 11,462,024)

^b^Mean of 684, 427 and 489 day hospitalizations in 2016, 2017 and 2018, respectively, corrected for the Belgian population mid-year 2019 (n = 11,462,024). For 2018 the number of chirurgical day hospitalizations is missing as it was smaller than 5

Per patient, the total cost, including ambulatory care, hospital care, travel expenses and productivity losses, corresponded to a mean of €193 for EM patients and €5,148 for disseminated/late LB patients. Regarding the latter, €1,778 per patient was related to hospitalizations (direct medical costs and productivity losses due to classical hospitalization or day hospitalization).

## Discussion

In order to estimate the economic burden of LB in Belgium, the current study used different data sources (a prospective cohort study and national hospital registries) which allowed the inclusion of direct medical costs, direct non-medical costs and indirect non-medical costs associated with this multisystem infectious disease. Based on an incidence approach, societal perspective and 2019 euros, the total annual cost associated with LB in Belgium was estimated at €5.59 million with a 95% UI between €3.82 and €7.98 million. Direct medical costs and productivity losses accounted for the majority of the costs, 49.8% and 46.4% of the total, respectively. Despite the much lower incidence of disseminated/late LB manifestations compared to EM, the former accounted for more than half of the total national LB costs per year (62%), because of its high mean cost of €5,148 (95% UI 3,091–7,911) per patient compared to a mean of €193 (95% UI 121–284) per EM patient. While the mean ambulatory direct medical cost was almost twice as high for patients with PTLDS after EM compared to EM patients without PTLDS, patients with PTLDS after disseminated/late LB incurred only higher productivity losses relative to patients without PTLDS after disseminated/late LB but no higher direct medical costs. The latter is unexpected, yet, these results need to be interpreted with caution as the sample sizes for the groups with PTLDS in the prospective cohort study were very small, causing uncertainty to be high. Although the majority of direct medical costs were reimbursed (75.5%), patients still pay €43.9 (EM) or €286 (Disseminated/late) of direct medical costs themselves. In addition, all direct non-medical costs are payed by the patient (i.e. travel expenses: €5 (EM) or €190 (Disseminated/late) and paid help: €50.9 (Disseminated/late)), making the cost to the patient still quite high. Furthermore, depending on the employment status and duration of sick leave there can be loss in salary.

Comparison of the current results with LB cost studies performed elsewhere is challenging as there is heterogeneity in the methods used, the patient populations studied and the cost data collected, as well as in the healthcare systems in place in the countries concerned. In Europe, some older cost studies have been performed for LB in Scotland (1999) [25], for LNB patients in Sweden (2000–2005) [26] and for hospitalized patients in Germany (2008–2011) [27]. Similar to our study, van den Wijngaard et al. (2017) estimated LB costs for the Netherlands, including direct medical and indirect non-medical costs [28]. For EM, a mean ambulatory cost of €136 was estimated (amount adjusted to 2019 price level [29, 30]), which is lower than our mean estimate of €193 in EM patients (including 5.9% patients with PTLDS [31]). Note that in contrast to the Dutch study, our study included OTC medication, laboratory testing and travel expenses with a cost of €40. The mean total cost for disseminated LB in the Dutch study, including ambulatory and hospital costs as well as costs for productivity losses, was estimated at €6,327 (inflated [29]), which is higher compared to the mean of €5,148 in our study (including 20.9% patients with PTLDS). The total costs for patients with persisting symptoms in the Netherlands were estimated as a separate category, which represented a substantial amount of €6,361 per patient (inflated [29]), on top of the costs for EM or disseminated LB. Out of these, €1,362 concerned ambulatory direct medical costs, which is much higher than the additional ambulatory direct medical costs found for PTLDS patients in our study (Table 2), yet sample size for the latter was very small. Also, patients with persisting symptoms were defined differently than our PTLDS patients and were included based on a GP diagnosis, so it may be that these were more seriously ill patients; the duration of persisting symptoms in that study was longer (4.6 years). Furthermore, some cost studies have also been performed in the US, but comparisons with non-US estimates are problematic due to fundamental differences in pricing and healthcare organization, as well as differences in clinical manifestations, with a higher proportional occurrence of more costly LA cases in the US [32–35]. Further research on the costs of patients with PTLDS seems warranted.

Comparison of our study results with the cost-of-illness of infectious diseases other than Lyme borreliosis in Belgium is also not straightforward as both costs and incidence data must be taken into account. For several infectious diseases, the average cost per case is lower (e.g., influenza [36, 37], rotavirus [38]) or similar (e.g., herpes zoster [39]) than the cost of LB, but due to the higher incidence of these infections in Belgium, their total national cost is much higher than that of LB. For example, for influenza, mean ambulatory direct costs have been estimated at €51–64 per case and hospitalization cost at €2,599, which, with an estimated 400 000 cases [36, 37], leads to a national cost of more than €30 million (2011–2012, indirect costs not included). Also for human papilloma virus (HPV), where mean costs per case and incidences differ largely by outcome (e.g., cervical cancer vs. anogenital warts), overall national costs are higher than LB [40]. Some other infections, have a lower direct cost due to a low incidence (e.g. hepatitis A [41]) or due to a low cost per case (e.g. Varicella [39]).

It is important to note that, in the current study, only costs for patients with clinical LB manifestations, either EM or laboratory confirmed disseminated/late LB, were included [31]. As such, costs for consultations for a tick bite without LB development were not counted even though they might take place due to worries about LB. Based on data from the SGP network (2015─2017), the annual number of GP consultations for tick bites can be estimated at 19,422 (95% UI 16,384–22,523), which corresponds to an estimated cost of €510,806 (95% UI 430,901–592,368) (productivity losses not counted). For the same reason, only laboratory tests performed in patients with clinical LB manifestations were included, however, many more LB serological tests are performed each year in Belgium. In the period 2015─2017, in ambulatory care only, an annual mean of 220,270 IgG EIA tests and 11,405 IgG Immunoblots were performed on blood, indicating a low positivity rate (5.18%) for the EIA (first-tier test) as immunoblot is recommended to only be performed when EIA is positive or equivocal. This indicates a potential over-use of these tests without benefits for patient management. Note that these serological tests are not advised for EM diagnosis, as in this early disease stage sensitivity is too low. In addition, tests do not differentiate between active and past infection and are not useful to follow-up antibiotic treatment success [42]. A high economic burden of several million euros related to these tests can be expected; the mean annual cost for all LB serological tests (IgG, IgM, EIA, Immunoblot, on blood, on CSF) in 2015─2017 equaled €1,092,801 and in addition a forfeit by analysis by patient (not by test) of €19.84─€30.5, depending on the number of tests performed, applies. More research into these costs and the reasons why these high numbers of tests are carried out is therefore of great importance. Also, no costs for prophylaxis treatment were included in the current study.

Since there are yearly fluctuations in the number of LB cases, this study used mean incidence estimates for a period of three years. Nevertheless, this incidence estimate was probably not representative for the number of LB cases in 2019, as 2019 was a year with an exceptionally low number of tick bites, probably due to extreme dry and warm periods during the summer [43]. It is therefore expected that the total costs for the year 2019 would be lower than the current cost estimates based on the LB incidence 2015─2017, but the latter are expected to be more representative for an average year.

As is the case for many healthcare cost data, most costs were skewed with a few patients incurring much higher costs than the majority of patients, causing estimated mean costs to be higher than median costs (Additional file 2). For example, for ambulatory productivity loss the median cost equaled €0 in EM patients and €656 for disseminated/late LB patients compared to a mean of €63.9 and €2,624, respectively. Yet, as is appropriate for cost-of-illness and cost-effectiveness analysis, we focused on mean costs, and extrapolated these to the population level to produce our overall cost burden estimates [44].

There are some limitations to the current study. First, for the ambulatory cost calculation, no children were included, hence the same costs are assumed as for adults. Second, as data are based on the incidence estimates 2015─2017 published by Geebelen et al. (2019), limitations mentioned there also apply to the current estimates [4]. As a possible underestimation of the proportion of disseminated/late LB manifestations was suspected, national costs for this group in the current study, can also be underestimated. Third, the sample size of the disseminated/late LB group and the group of patients with PTLDS in the prospective cohort study, used for ambulatory cost calculation, was small and uncertainty in the results high. Further research into their costs is needed. Fourth, hospitalizations for LB were selected based on ICD-10-coding of LB, which mainly serves for financing and was not necessarily developed for surveillance purposes. Quality of the coding could not be checked. Since there is no specific ICD-10-CM code for EM or PTLDS, all hospital costs were allocated to the disseminated/late LB group. Although no hospital costs are expected due to the mild nature of the symptoms of EM, they may be present in exceptional cases. Fifth, while a societal perspective was taken, it was not possible to include all costs that might have occurred; no ambulatory laboratory testing other than LB serology, no medical acts for emergency visits and not all non-reimbursed costs and supplements for hospitalizations could be included. On the other hand, for hospitalizations, costs for all types of medication, laboratory testing or medical acts were included, comprising also costs for comorbidities not related to LB (e.g. blood pressure medication). Finally, for EM or disseminated/late LB patients, no costs exceeding 6 months post diagnosis were included, which could result in an underestimation of the true cost of LB in Belgium. Nevertheless, after a longer period it becomes more difficult (also for the patient) to relate specific costs to the disease.

In conclusion, we provide for the first time, a comprehensive assessment of the costs related to LB in Belgium. As EM and disseminated/late LB are estimated to cause about 40% and 60% of the total national costs, respectively, both measures to prevent EM, such as prevention of tick bites and fast removal of ticks and prevention of dissemination of disease, such as informing GPs and the public on EM to improve fast treatment, are essential. Ideally, a vaccine will be developed that prevents tick bites, hence prevents LB (all manifestations), but also prevents transmission of other tick-borne pathogens. The current cost estimation can serve as an input in future cost-effectiveness analyses of such vaccines or other pharmaceutical and non-pharmaceutical interventions. While patients with PTLDS seem to have somewhat higher costs compared to patients without PTLDS this needs further research.

## Supplementary Information

**Additional file 1: **Unit prices ambulatory care (2019). **Table S1.** Consultations. **Table S2.** Laboratory tests ambulatory care. **Table S3.** Antibiotic therapy. **Table S4.** Other ambulatory costs.

**Additional file 2: **Estimated median costs per patient. **Table S5.** Estimated median ambulatory cost per patient, expressed in 2019 euros, for the different manifestation groups of Lyme borreliosis in the prospective cohort study (HUMTICK), 2016─2020, Belgium. Mean and 95% uncertainty intervals of the bootstrap distribution of the median. **Table S6.** Estimated median costs per classical hospitalization and day hospitalization, 2016 euros converted to 2019 euros, Belgium. Mean and 95% uncertainty intervals of the bootstrap distribution of the median.

**Additional file 3: **Incidence number of cases and total costs for LB in Belgium for the healthcare insurance system or patient. **Table S7.** Incidence number of cases and total costs for LB in Belgium for the healthcare insurance system or patient. Total costs and 95% uncertainty interval.

## Data Availability

The datasets generated and analyzed during the current study are not publicly available as restrictions apply to the availability of these data. Part of the data can be made available from the corresponding author on reasonable request if compliant with the approvals received for the data collection.

## Abbreviations

EM: Erythema migrans
LNB: Lyme neuroborreliosis
LA: Lyme arthritis
ACA: Acrodermatitis chronica atrophicans
PTLDS: Post-treatment Lyme disease syndrome
IDSA: Infectious Disease Society of America
SGP: Sentinel general practices
GP: General practitioner
KCE: Belgian Health Care Knowledge Centre
MCD: Minimal Clinical Data registry
MFD: Minimal Financial Data registry
ICD-10-CM: International Statistical Classification of Diseases—10th revision—Clinical Modification
UI: Uncertainty interval
OTC: Over-The-Counter
