# Supplementary material for: Lyme borreliosis in Belgium: a cost-of-illness analysis
Source: BMC Public Health. 2022 Nov 28;22:2194. doi: 10.1186/s12889-022-14380-6 (PMC9703731; doi:10.1186/s12889-022-14380-6)
Supplement: Supplementary file 1 — Additional file 1: Unit prices ambulatory care (2019). Table S1. Consultations. Table S2. Laboratory tests ambulatory care. Table S3. Antibiotic therapy. Table S4. Other ambulatory costs. [file 12889_2022_14380_MOESM1_ESM.docx]

**Additional files**

**Contents:**

Additional file 1: Unit prices ambulatory care (2019). Table S1. Consultations

- Table S2. Laboratory tests ambulatory care
- Table S3. Antibiotic therapy
- Table S4. Other ambulatory costs

**Additional file 1: Unit prices ambulatory care (2019)**

**Table S1. Consultations**

Prices for accredited care providers applicable on 01/01/2019 and reimbursements without increased allowance were counted. For some specialists a higher reimbursement is foreseen for the first consultation when referred to by a GP which was assumed to be the case. For emergency visits, the price without referral by a GP was counted. No additional services were counted unless explicitly reported by the patient (e.g. scan under other expenses) and no additional weekend/evening/night tariffs were counted as this information was not available for all consultations.

| **Reimbursed care providers** | **Total fee** | **Reimbursed** | **Patient fee** | **Source** |
| --- | --- | --- | --- | --- |
| **GP^1^** | €26.27 | €22.27 | €4 | [1] |
| **Neurologist** | €59.96 | €47.96  *€52.96 | €12.00  *€7.00 | [1] |
| **Rheumatologist** | €59.05 | €47.05  *€52.05 | €12.00  *€7.00 | [1] |
| **Internal medicine/** **infectious disease specialist** | €46.50 | €34.50  *€39.50 | €12.00  *€7.00 | [1] |
| **Dermatologist** | €33.67 | €21.67  *€26.67 | €12.00  *€7.00 | [1] |
| **Cardiologist** | €39.38 | €27.38  *€32.38 | €12.00  *€7.00 | [1] |
| **Gastro-enterologist** | €39.38 | €27.38  *€32.38 | €12.00  *€7.00 | [1] |
| **Physical medicine** | €29.04 | €17.04  *€22.04 | €12.00  *€7.00 | [1] |
| **Radiologist**  **Consultation +**  **Fee prescription (CT/MRI)**  **Fee prescription (echo)** | €27.31 +  €44.70  €12.59 | €19.87 +  €44.70  €12.59 | €7.44 +  €0  €0 | [2] |
| **Orthopedic specialist** | €26.27 | €14.27  *€19.27 | €12.00  *€7.00 | [1] |
| **Ophthalmologist** | €26.27 | €14.27  *€19.27 | €12.00  *€7.00 | [1] |
| **Physiotherapist** | €22.26 | €16.37 | €5.89 | [3] |
| **Psychologist^2^** | €60.00 | €49.00 | €11.00 | [4] |
| **Emergency department (ED)^3^** | €34.90 | €13.80 | €21.10 | [5] |
| **Alternative therapists^4^:** Homeopath^5^, osteopath, chiropractor | €50 | €0 | €50^6^ | [6–8] |

^1^ Consultation in the consulting room with global medical record

^2^ Psychological session of 60 minutes

^3^ Mean price of a consultation with a specialist in emergency medicine, a specialist in acute medicine, a specialist and a physician with an acute medicine license (all accredited), no referral by GP counted for reimbursement

^4^ No reimbursement counted as it is not standard and depends on the health insurance fund of the patient and the alternative therapist consulted whether or not part of consultations are reimbursed

^5^ A primary consult can be more expensive, for this study it is assumed that the patients have consulted a homeopath before

^6^ The mean of the price range provided by the Belgian professional association of osteopathy in 2019 [8] and the price ranges for a consultation with a homeopath or chiropractor in Belgium reported by De Gendt et al. (2010 & 2011) [6, 7]─ adding inflation of prices between 2009 and 2019

**Table S2. Laboratory tests ambulatory care**

| **Type cost** | **Total fee** | **Reimbursed** | **Patient fee** | **Source** |
| --- | --- | --- | --- | --- |
| **Elisa IgG** | €1.95 | €1.95 | €0 | [10] |
| **Elisa IgM** | €2.34 | €2.34 | €0 | [10] |
| **Blot IgG** | €9.38 | €9.38 | €0 | [10] |
| **Blot IgM** | €9.38 | €9.38 | €0 | [10] |
| **Forfait^1^** | €19.84 – 36.37 | €19.84 –23.41 | €0-12.96 | [10] |

*Cerebrospinal fluid testing is counted under (day) hospitalizations.*

^1^ Amount dependent on number of tests performed

**Table S3. Antibiotic therapy**

|  | **N tablets** | **Total fee** | **Reimbursed** | **Patient fee** | **Source** |
| --- | --- | --- | --- | --- | --- |
| **Doxycycline 100 mg** | 10 | €6.86 | €5.32 | €1.54 | [9] |
| **Doxycycline 200 mg** | 10 | €8.54 | €5.78 | €2.76 | [9] |
| **Amoxicilline 500 mg** | 16 | €6.76 | €5.3 | €1.46 | [9] |
| **Amoxicilline 500 mg** | 30 | €11.85 | €6.82 | €5.03 | [9] |
| **Amoxicilline 1000 mg** | 20 | €12.73 | €7.1 | €5.63 | [9] |
| **Amoxicilline 1000 mg** | 24 | €13.63 | €7.37 | €6.26 | [9] |
| **Cefuroxim 500 mg** | 10 | €10.63 | €6.43 | €4.2 | [9] |
| **Cefuroxim 500 mg** | 20 | €15.96 | €8.1 | €7.86 | [9] |
| **Amoxi/clav 500/125 mg** | 16 | €10.11 | €6.27 | €3.84 | [9] |
| **Amoxi/clav 500/125 mg** | 30 | €13.65 | €7.38 | €6.27 | [9] |
| **Amoxi/clav 875/125 mg** | 20 | €13.99 | €7.48 | €6.51 | [9] |
| **Tetralysal 300** | 28 | €19.47 | €9.21 | €10.26 | [9] |

*Ceftriaxone treatment is counted under (day) hospitalizations*

**Table S4. other ambulatory costs**

| **Type cost** | **Total fee** | **Reimbursed** | **Patient fee** | **Source** |
| --- | --- | --- | --- | --- |
| **CT-scan knee** | €98.65 | €96.17 | €2.48 | [2] |
| **MRI** | €52.44 | €49.96 | €2.48 | [2] |
| **Echo lower extremities** | €40.13 | €37.65 | €2.48 | [2] |

**Additional file 2: Estimated median costs per patient**

**Table S5. Estimated median ambulatory cost per patient, expressed in 2019 euros, for the different manifestation groups of Lyme borreliosis in the prospective cohort study (HUMTICK), 2016─2020, Belgium. Mean and 95% uncertainty intervals of the bootstrap distribution of the median.**

|  | **Unit cost** | **(i) Erythema migrans** | | | **(ii) Disseminated/late LB** | | |
| --- | --- | --- | --- | --- | --- | --- | --- |
|  |  | **No PTLDS** | **PTLDS** | **All** | **No PTLDS** | **PTLDS** | **All** |
| **Nb pts in cohort** |  | **102** | **6** | **108** | **12** | **3** | **15** |
| **Total direct medical costs** |  | **€52.5 (45.9-60.6)^1^** | **€146.4 (53.9-433.1)^1^** | **€53.7 (46.9-63.0)^1^** | **€421 (212-715)** | **€470 (262-723)** | **€429 (212-723)** |
| **Consultations** |  | **€26.3 (26.3-26.3)** | **€80.8 (26.3-182.4)** | **€26.3 (26.3-26.3)** | **€351 (169-544)** | **€305 (85.3-382)** | **€ 346 (156-526)** |
| GP | *€26.3* | €26.3 (26.3-26.3) | €44.4 (26.3-78.8) | €26.3 (26.3-26.3) | €87.4 (59.1-158) | €116 (0-158) | €94.3 (52.5-158) |
| Specialist^2^ | *€26.3-60.0* | €0 (0-0) | €3.3 (0-46.5) | €0 (0-0) | €135 (97.5-179) | €116 (85.3-139) | €129 (85.3-179) |
| Emergency^3^ | *€34.9* | €0 (0-0) | €0 (0-0) | €0 (0-0) | €30.6 (0-34.9) | €43.9 (0-105) | €32 (0-34.9) |
| Alternative^4^ | *€50* | €0 (0-0) | €3.8 (0-50) | €0 (0-0) | €16.6 (0-75) | €12.9 (0-50) | €11.2 (0-50) |
| Others^5^ | *€22.3-60.0* | €0 (0-0) | €2.5 (0-33.4) | €0 (0-0) | €3.4 (0-44.5) | €0 (0-0) | €0.4 (0-0) |
| **Medication** |  | **€18.8 (13.7-21.2)** | **€68.7 (14.5-250.6)** | **€19.3 (17.1-21.3)** | **€70.3 (36.7-133)** | **€161.4 (65.8-341)** | **€79.7 (41.2-164)** |
| Antibiotics at T0 |  | €16.3 (13.7-18.6) | €15 (11.1-20.6) | €16 (13.7-18.6) | €3 (0-27.4) | €26.7 (0-41.2) | €5.4 (0-34.3) |
| Prescription |  | €0 (0-0) | €4.3 (0-32.1) | €0 (0-0) | €24.1 (0-47.6) | €47.4 (13.4-75.8) | €29.1 (0-49.4) |
| Over the counter |  | €0 (0-0) | €38.9 (0-212) | €0 (0-0) | €25.8 (7.7-71.5) | €83.3 (0-294) | €23.4 (12.1-90.1) |
| **LB serology** |  | ***€0 (0-0)^6^*** | ***€0 (0-0)^6^*** | ***€0 (0-0)^6^*** | **€0.3 (0-0)^7^** | **€15.5 (0-59.4)^7^** | **€0.3 (0-0)^7^** |
| **Other^8^** |  | **€0 (0-0)** | **€0 (0-0)** | **€0 (0-0)** | **€ 0 (0-0)** | **€ 0 (0-0)** | **€0 (0-0)** |
| **Indirect non-medical costs** |  |  |  |  |  |  |  |
| **Ambulatory^9^** | *€40.5/hr* | **€0 (0-0)** | **€15.1 (0-213)** | **€0 (0-0)** | **€332 (0-2,946)** | **€3,578 (0-5,604)** | **€654 (0-4,427)** |

^1^ LB serology not included as not available by patient

^2^ Neurologist, rheumatologist, infectious disease specialist, dermatologist, gastroenterologist, orthopedist, radiologist (consult + forfait by prescription)

^3^ Visit and care included only (no medication/performances); price visit without referral by a GP, on a weekday and care provided by a specialist in emergency medicine

^4^ Homeopath, osteopath, chiropractor

^5^ Physiotherapist, psychologist

^6^ Based on SGP data 2015─2017: at diagnosis or in the first 2 months after, ELISA (1st tier test) was performed in 39.7% and Immunoblot (2nd tier test) in 19.0% of EM cases with available information (n=348), no differentiation could be made between patients without or with PTLDS

^7^ Only includes testing performed in patients that were not hospitalized and did not have Lyme neuroborreliosis (n=3), as the latter needs cerebrospinal fluid testing, all tests in these patients and hospitalized patients were expected to be performed during (day-)hospitalization.

^8^ Scans: CT-scan, MRI or echo in ambulatory patient

^9^ Productivity losses taking into account part-time work in the cohorts

Nb: Number; LB: Lyme borreliosis; PTLDS: Post-treatment Lyme disease syndrome; SGP: sentinel general practices; GP: general practitioner; EM: erythema migrans

**Table S6. Estimated median costs per classical hospitalization and day hospitalization, 2016 euros converted to 2019 euros, Belgium. Mean and 95% uncertainty intervals of the bootstrap distribution of the median.**

|  | **Median unit**  **cost** | **Cost per hospital stay** |
| --- | --- | --- |
|  |  | **Mean (95% UI)** |
| **Overnight hospital stays (n=286)** | |  |
| **Total direct medical costs** |  | **€2,637 (2,295-3,030)** |
| **100 % day cost** | **€525** | **€1704.1 (1453-2120.2)** |
| **Medication** |  | **€122 (117-127)** |
| Lump sum per admission | €95.7 | €90 (88.1-92.7) |
| Lump sum per day^1^ | €0.62 | €2.1 (1.9-2.5) |
| Antibiotics^2^ |  | €4.4 (1.0-7.3) |
| Pain killers |  | €0 (0-0) |
| Others |  | €12.5 (9.9-15.4) |
| **Laboratory tests** |  | **€64 (59.2-71.6)** |
| LB serology |  | € 7.6 (6.1-11.8) |
| Others |  | € 52.1 (45.9-58.6) |
| **Medical acts** |  | **€667 (589.7-733.8)** |
| Clinical biology |  | €163 (153-180) |
| Permanence, examinations |  | €167 (154-186) |
| Medical imaging |  | €160 (135-175) |
| Others^3^ |  | €113 (101-132) |
| **Implants^4^** |  | **€0 (0-0)** |
| **Radiopharmaceuticals** |  | **€0 (0-0)** |
| **Indirect non-medical cost (productivity loss)^5^** | **€40.5/hr** | **€269 (178-356)** |
|  |  |  |
| **Day hospital (n=613)** | | |
| **Total direct medical costs** |  | **€14.4 (14-15.6)** |
| **Indirect non-medical cost (productivity loss)^5^** | **€40.5/hr** | **€29.8** |

*^1^ Lump sum per day: forfait for reimbursed medicines, charged to the patient by day even if such medicines have not been used.*

^2^ *Antibiotics probably related to Lyme borreliosis: Ceftriaxon, Doxycycline, Amoxicilline, Clarithromycine, cefuroxime, azithromycine, cefotaxim, ampicilline cefepime, cefazoline and flucloxacilline.*

*^3^ Including internal medicine, revalidation, surgery, night/weekend supplements and others.*

*^4^ Mainly catheter*

*^5^ Based on* ***median*** *bootstrapped days of stay (<65 yrs olds) * proportion hospitalizations < 65 yrs old * proportion of < 65 yrs olds working *proportion working days *7.6 hours * €40.5. For day hospitalizations, the number of days was multiplied with 0.5 as half a day of sick leave was counted.*

The estimated mean of the median travel expenses related to ambulatory care or hospitalization equaled €0 (95% UI 0-0) in EM patients and €129 (0-219) in disseminated/late LB patients. Within the EM group, median costs were higher in patients with PTLDS (€6.4, 95% UI 0-46) compared to no PTLDS (€0, 95% UI 0-0). For disseminated/late LB, costs were lower for patients with PTLDS (€91.9, 95% UI 0-219) than no PTLDS (€155, 95% UI 0-434), but the number of patients in the disseminated/late LB group and PTLDS groups were low.

**Additional file 3:** **Incidence number of cases and total costs for LB in Belgium for the healthcare insurance system or patient.**

**Table S7: Incidence number of cases and total costs for LB in Belgium for the healthcare insurance system or patient. Total costs and 95% uncertainty interval.**

|  | **(i) Erythema migrans** | **(ii) Disseminated/late LB** | **Total** |
| --- | --- | --- | --- |
| **Nb cases** | **11,168 (9,417-12,954)** | **673 (519-867)** | **11,840 (9,976-13,732)** |
| **Nb hospitalizations** | **0** | **248** | **248** |
| **Nb day-hospitalizations** | **0** | **539** | **539** |
| **Direct medical costs** | **Ins.: €889,769 (650,710-1,212,840)  Pt.: €490,069 (269,825-790,456)** | **Ins.: €1,214,884 (1,069,390-1,385,462)  Pt.: €191,617 (101,720-333,853)** | **Ins.: €2,104,653 (1,800,383-2,474,597)  Pt.: €681,685 (425,171-1,021,477)** |
| Ambulatory | Ins.: €889,769 (650,710-1,212,840)  Pt.: €490,069 (269,825-790,456) | Ins.: €184,984 (129,145-256,279)  Pt.: €161,981 (72,453-303,738) | Ins.: €1,074,753 (810,758-1,418,809)  Pt.: €652,050 (395,997-991,616) |
| Hospital | €0 | Ins.: €1,005,719 (870,347-1,158,271)  Pt.: €26,606 (23,041-30,630) | Ins.: €1,005,719 (870,347-1,158,271)  Pt.: €26,606 (23,041-30,630) |
| Day-hospital | €0 | Ins.: €24,182 (16,364-34,492)  Pt.: €3,029 (2,499-3,941) | Ins.: €24,182 (16,364-34,492)  Pt.: €3,029 (2,499-3,941) |
| **Direct medical costs per patient** | **Ins.: €79.7 (63.1-102.8)**  **Pt.: €43.9 (25.1-68.1)** | **Ins.: €4632 (2615-7430)**  **Pt.: €286 (160-476)** | **Ins.: €179 (152-209)**  **Pt.: €57.6 (37.5-82.1)** |

Nb : Number ; Ins.: Insurance ; Pt.: Patient

**References**

1. Institut National d’Assurance Maladie-Invalidité (I.N.A.M.I). Tarifs ; médecins - consultations et visites ; 01-01-2019 ; A. 2019. https://www.riziv.fgov.be/SiteCollectionDocuments/tarif_medecins_partie01_20190101.pdf. Accessed 22 Dec 2021.

2. Institut National d’Assurance Maladie-Invalidité (I.N.A.M.I). Tarifs ; médecins - imagerie médicale ; 01-01-2019. 2019. https://www.riziv.fgov.be/SiteCollectionDocuments/tarif_medecins_partie04_20190101.pdf. Accessed 22 Dec 2021.

3. Institut National d’Assurance Maladie-Invalidité (I.N.A.M.I). Tarifs ; Kinésithérapeutes ; 01-01-2019. 2019. https://www.riziv.fgov.be/SiteCollectionDocuments/tarif_kinesitherapeutes_20190101.pdf. Accessed 22 Dec 2021.

4. Institut National d’Assurance Maladie-Invalidité (I.N.A.M.I). Tarifs des séances de psychologie de première ligne à partir du 1-1-2019. https://www.riziv.fgov.be/SiteCollectionDocuments/tarif_ppl_orthopedagogue_psychologue_clinique_20190101.pdf. Accessed 22 Dec 2021.

5. Institut National d’Assurance Maladie-Invalidité (I.N.A.M.I). Tarifs ; médecins - consultations et visites ; 01-01-2019 ; B. 2019. https://www.riziv.fgov.be/SiteCollectionDocuments/tarif_medecins_partie02_20190101.pdf. Accessed 22 Dec 2021.

6. De Gendt T, Desomer A, Goosens M, Hanquet G, Léonard C, Mertens R, et al. Stand van zaken van de homeopathie in België. Health Services Research (HSR). Brussel: Federaal Kenniscentrum voor de Gezondheidszorg (KCE). 2011.

7. De Gendt T, Desomer A, Goossens M, Hanquet G, Léonard C, Mélard F, et al. Stand van zaken voor de osteopathie en de chiropraxie in België. Health Services Research (HSR). Brussel: Federaal Kenniscentrum voor de Gezondheidszorg (KCE). 2010.

8. www.osteopathie.be. Kosten en vergoeding. 2019. https://osteopathie.be/nl/osteopathie-ik-ben-patient/kosten-vergoeding-osteopathische-zorg/. Accessed 25 Nov 2019.

9. Belgian Centre for Pharmacotherapeutic Information. Répertoire commenté des médicaments. 2019. http://www.cbip.be/fr/start. Accessed 30 Dec 2019.

10. Institut National d’Assurance Maladie-Invalidité (I.N.A.M.I). Tarifs ; médecins - biologie clinique ; 01-01-2019. 2019. https://www.riziv.fgov.be/SiteCollectionDocuments/tarif_medecins_partie05_20190101.pdf. Accessed 22 Dec 2021.
