# Supplementary material for: Lyme borreliosis in Belgium: a cost-of-illness analysis
Source: BMC Public Health. 2022 Nov 28;22:2194. doi: 10.1186/s12889-022-14380-6 (PMC9703731; doi:10.1186/s12889-022-14380-6)
Supplement: Supplementary file 2 — Additional file 2: Estimated median costs per patient. Table S5. Estimated median ambulatory cost per patient, expressed in 2019 euros, for the different manifestation groups of Lyme borreliosis in the prospective cohort study (HUMTICK), 2016─2020, Belgium. Mean and 95% uncertainty intervals of the bootstrap distribution of the median. Table S6. Estimated median costs per classical hospitalization and day hospitalization, 2016 euros converted to 2019 euros, Belgium. Mean and 95% uncertainty intervals of the bootstrap distribution of the median. [file 12889_2022_14380_MOESM2_ESM.docx]

Additional file 2: Estimated median costs per patient

- Table S5. Estimated median ambulatory cost per patient, expressed in 2019 euros, for the different manifestation groups of Lyme borreliosis in the prospective cohort study (HUMTICK), 2016─2020, Belgium. Mean and 95% uncertainty intervals of the bootstrap distribution of the median.
- Table S6. Estimated median costs per classical hospitalization and day hospitalization, 2016 euros converted to 2019 euros, Belgium. Mean and 95% uncertainty intervals of the bootstrap distribution of the median.
