# Supplementary material for: Lyme borreliosis in Belgium: a cost-of-illness analysis
Source: BMC Public Health. 2022 Nov 28;22:2194. doi: 10.1186/s12889-022-14380-6 (PMC9703731; doi:10.1186/s12889-022-14380-6)
Supplement: Supplementary file 3 — Additional file 3: Incidence number of cases and total costs for LB in Belgium for the healthcare insurance system or patient. Table S7. Incidence number of cases and total costs for LB in Belgium for the healthcare insurance system or patient. Total costs and 95% uncertainty interval. [file 12889_2022_14380_MOESM3_ESM.docx]

Additional file 3: Incidence number of cases and total costs for LB in Belgium for the healthcare insurance system or patient.

- Table S7: Incidence number of cases and total costs for LB in Belgium for the healthcare insurance system or patient. Total costs and 95% uncertainty interval.
